# Surveillance of Vector-Borne Zoonotic Diseases in South Korea: Uncovering Novel Pathogen Carriers Among Rodents and Mites Nationwide

**DOI:** 10.1155/2024/5544660

**Published:** 2024-11-08

**Authors:** Beoul Kim, You-Jeong Lee, Hee Il Lee, Dongmi Kwak, Min-Goo Seo

**Affiliations:** ^1^College of Veterinary Medicine and Institute for Veterinary Biomedical Science, Kyungpook National University, 80 Daehak-ro, Buk-gu, Daegu 41566, Republic of Korea; ^2^Division of Vectors and Parasitic Diseases, Korea Disease Control and Prevention Agency, 187 Osongsaenmyeong2-ro, Osong-eup, Heungdeok-gu, Cheongju 28159, Republic of Korea

**Keywords:** Lyme disease, mite, Q fever, scrub typhus, SFTS, wild rodent

## Abstract

Wild rodents and their ectoparasites are known reservoirs for various zoonotic pathogens, highlighting the need for detailed studies into their roles in disease transmission. Our research investigated the spatial distribution of rodents and their ectoparasites to better understand the epidemiology of vector-borne zoonotic diseases (VBZDs), including severe fever with thrombocytopenia syndrome (SFTS), Lyme disease, Q fever, and scrub typhus. We analyzed samples from 540 rodents and 6785 mites, detecting the presence of *Borrelia* spp., the causative agent of Lyme disease, in 0.9% of rodents and SFTS virus (SFTSV) in 1.0%. In mites, *Borrelia* spp. and *Orientia tsutsugamushi*, the bacteria causing scrub typhus, were detected in 0.3% of samples each. Phylogenetic analysis identified the SFTSV sequence as type B3, the *Borrelia* spp. sequence as *B. afzelii*, and the *O. tsutsugamushi* sequence as Karp-related. Notably, SFTSV was detected for the first time in mites in South Korea, and *B. afzelii* was found in mites for the first time globally. These findings emphasize the critical need for continuous analysis of VBZDs to anticipate future trends and develop a comprehensive monitoring system. Further research into the rodent and mite populations in South Korea is essential to fully assess the potential risks of VBZDs.

## 1. Introduction

Zoonotic diseases, which are illnesses caused by pathogens transmitted between animals and humans, pose an increasing threat to public health. Recent data from the Korea Disease Control and Prevention Agency (KDCA) indicate a consistent emergence or rise in vector-borne zoonotic diseases (VBZDs) in South Korea over the past decade, including severe fever with thrombocytopenia syndrome (SFTS), Lyme disease, Q fever, and scrub typhus [1]. In the context of globalization, characterized by climate change, and the increased international movement of people and animals, the rise of these diseases underscores the urgency of prioritizing their prevention and management [2]. The One Health concept, which integrates human, animal, and environmental ecosystems, is critical in this regard. Despite extensive nationwide surveys by the KDCA on the distribution of chiggers, particularly concerning diseases like scrub typhus, there remains a significant disparity in surveillance and research efforts on other VBZDs transmitted by mites compared to those transmitted by ticks [3]. Additionally, wild rodents serve as reservoirs for various zoonotic pathogens, emphasizing the need for simultaneous investigations into both the captured rodents and their attached ectoparasites to assess their potential role in disease transmission. While VBZDs such as SFTS [4], Lyme disease [5–7], and Q fever [8–10] have been detected in rodent populations globally, research focused on VBZDs within domestic rodent populations in South Korea is notably scarce, except for studies on scrub typhus in mites [11–13]. Furthermore, although active research on VBZDs in humans and animals is prevalent internationally, South Korea's domestic research landscape shows a lack of comparable studies. This study, therefore, conducted a comprehensive survey of VBZDs in domestic rodents and their vectors, specifically mites. The objectives of this survey are to compare and analyze infection rates, predict potential disease outbreaks, and establish an effective surveillance system.

## 2. Materials and Methods

### 2.1. Ethical Approval

The animal protocol employed in this study adhered to the scientific care guidelines and ethical procedures established by the Institutional Animal Care and Use Committee (IACUC) of the Korea Centers for Disease Control and Prevention (KCDC-093-18) and was approved by the committee. The rodent and mite samples, considered as biological resources, were sourced from the KDCA. Furthermore, this study was reviewed and approved in line with the ethical procedures and scientific care guidelines of the IACUC at Kyungpook National University (KNU 2022-0441).

### 2.2. Surveillance of Rodents

In collaboration with the Regional Center for Vector Surveillance against Climate Change, operated by the KDCA, wild rodents were captured using rodent traps at 16 national collection sites. Collections were made four times at each site during spring (March and April) and autumn (October and November) of 2022. Each site consisted of five environmental sampling points, each in different habitats: rice paddies, grasslands, reservoirs, waterways, and forests. Twenty Sherman live folding traps (3 × 3 × 9 in.; BioQuip, CA, USA) were placed at each sampling points, totaling 100 traps per site. Traps were baited with biscuits spread with peanut butter and placed 3–5 m apart. The traps were set before sunset and retrieved the following morning, which optimized the effectiveness of rodent capture. Upon capture, each rodent was morphologically identified to species level using a taxonomic key, and immediate euthanasia was performed via CO_2_ inhalation. Liver tissue samples were then collected and stored at −70°C for later analysis. These biological resources, provided by the KDCA, were utilized for experimental purposes.

**Figure 1:**
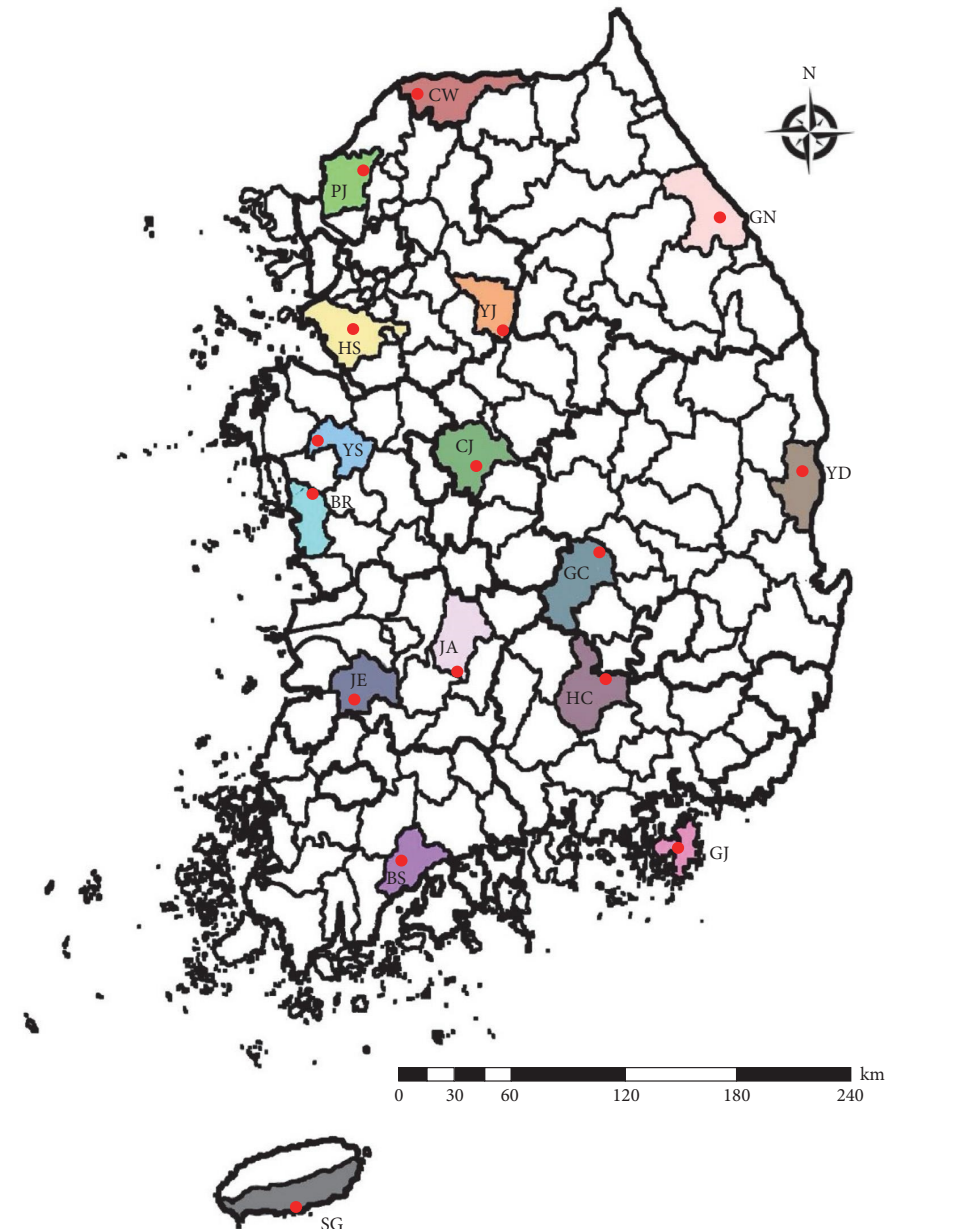
Sampling locations where rodents and mites were collected. Rodent traps are denoted by red dots. BR, Boryeong; BS, Boseong; CJ, Cheongju; CW, Cherwon; GC, Gimcheon; GJ, Geoje; GN, Gangneung; HC, Hapcheon; HS, Hwasung; JA, Jinan; JE, Jeongeup; PJ, Paju; SG, Seogwipo; YD, Yeongdeok; YJ, Yeoju; YS, Yesan.

The regions were classified as northern, central, southern, and Jeju Island (Figure 1). In the northern region, samples were collected from Hwaseong, Paju, Yeoju, Cheorwon, and Gangneung. In the central region, collections were made in Cheongju, Boryeong, and Yesan. In the southern region, samples were collected from Jeongeup, Boseong, Jinan, Geoje, Hapcheon, Gimcheon, and Yeongdeok. Samples from Jeju Island were collected in Seogwipo.

### 2.3. Surveillance of Mites

To collect external ectoparasites such as mites, the captured rodents were first euthanized and dissected to obtain liver tissue samples. Subsequently, each rodent was suspended by the tail end, upside down on a stick over a beaker filled with water, within a Petri dish. This setup allowed external parasites to fall onto the Petri dish during a 24-h incubation period. The mites collected were preserved in 70% ethanol for further analysis. These specimens, provided by the KDCA, were utilized for experimental purposes. From the total mites attached to each rodent, a representative sample of 1–30 mites was selected for analysis.

### 2.4. Molecular Detection of VBZD

For deoxyribonucleic acid (DNA) extraction, mite pools (1–30 mites per rodent) and rodent tissues were homogenized using a Precellys CK28-R Lysing kit (a bead tube designed for hard tissue homogenization; Bertin Technologies, Bretonneus, France) and Precellys evolution Homogenizer (Bertin Technologies). Genomic DNA was then extracted using the DNeasy Blood and Tissue Kit (Qiagen, Melbourne, Australia), according to the manufacturer's instructions.

To amplify pathogen-specific genes, species-specific primers were used as documented in the literature (Table 1). These primers targeted the S segment of the SFTS virus (SFTSV), the 5S−23S ribosomal RNA (rRNA) of *Borrelia* spp., the 56-kda gene of *Orientia tsutsugamushi*, and the 16s rRNA of *Coxiella burnetii*. Polymerase chain reaction (PCR) was conducted to detect *C. burnetii*, *Borrelia* spp., and *O. tsutsugamushi* using the AccuPower HotStart PCR Premix kit (Bioneer, Daejeon, South Korea) and to detect SFTSV using the AccuPower reverse transcription-polymerase chain reaction (RT-PCR) Premix kit (Bioneer). In our study, we included both positive and negative controls to ensure the validity of our PCR results. Specifically, a sample of *O. tsutsugamushi* detected in chigger mites [3], SFTSV detected in ticks [21], *C. burnetii* detected in pigs [16], and *B. afzelii* detected in ticks [22] from our previous studies in South Korea were used as positive controls. A negative control, which did not contain a DNA template, was also included to verify the absence of contamination.

### 2.5. DNA Sequencing and Phylogenetic Analysis

PCR products that tested positive for the aforementioned pathogens were sorted and sent to Macrogen (Seoul, South Korea) for sequencing, along with their corresponding primers. These positive samples underwent sequencing to ascertain their nucleotide sequences. Comparative analysis was conducted by aligning these sequences with those in the National Center for Biotechnology Information (NCBI) GenBank database for molecular genetic analysis. The multiple sequence alignment tool, CLUSTAL Omega (v. 1.2.1, Bioweb, Ferndale, WA, USA), was used to align and analyze the sequences obtained in this study with those previously reported in GenBank. After identification with BioEdit (v. 7.2.5, BioEdit, Manchester, UK), redundant sequences—unnecessary sequences—were eliminated from the aligned nucleotide sequences. Before performing phylogenetic analysis, sites with gaps or uncertain alignments were excluded. Phylogenetic analysis was performed using MEGA (v. 6.0, Mega software solutions, Madhurawadha, India) employing the Kimura two-parameter distance model and the maximum likelihood method.

### 2.6. Statistical Analysis

Statistical analyses were conducted using GraphPad Prism Version 5.04 (GraphPad Software Inc., La Jolla, CA, USA). For tables involving more than two variables, Pearson's chi-square test was employed, whereas Fisher's exact test was used for 2 × 2 tables. *p*-Values of ≤0.05 were considered statistically significant. Additionally, 95% confidence intervals (CIs) were computed for all estimates.

## 3. Results

### 3.1. Identification of Rodents

Upon identifying 540 rodents, they were classified into seven species: *Apodemus agrarius* (81.3%, 439/540), *Apodemus peninsulae* (0.9%, 5/540), *Craseomys regulus* (1.3%, 7/540), *Crocidura* spp. (11.9%, 64/540), *Micromys minutus* (3.9%, 21/540), *Microtus fortis* (0.5%, 3/540), and *Mogera robusta* (0.1%, 1/540) (Table 2). *A. agrarius* was the most prevalent species among the collected rodents, and it was the only species in which VBZDs, notably *Borrelia afzelii*, were detected, with a prevalence of 1.1% (5/439, 95% CI: 0.1–2.1). Rodents were collected from five distinct environments: reservoirs, forests, rice paddies, waterways, and grasslands. The distribution of captured rodents across these environments was as follows: 128 (23.7%) from forests, 92 (17.0%) from grasslands, 73 (13.5%) from rice paddies, 133 (24.6%) from waterways, and 110 (20.4%) from reservoirs.

### 3.2. Occurrence of VBZDs in Wild Rodents

A total of 6400 traps were deployed, capturing 540 rodents across 16 survey locations. Among the VBZDs, only *B. afzelii* was detected, with a prevalence of 0.9% (5/540, 95% CI: 0.1–1.7) (Tables 2 and 3). Regional analysis indicated that *B. afzelii* was found exclusively in the northern region, with a prevalence of 3.0% (2/67, 95% CI: 0–7.1) in Cheorwon and 5.6% (3/54, 95% CI: 0–11.7) in Gangneung. Seasonally, the prevalence of *B. afzelii* was significantly higher in spring, at 1.9% (5/265, 95% CI: 0.2–3.5, *p*=0.0222). Furthermore, all positive cases of *B. afzelii* (1.1%, 5/439, 95% CI: 0.1–2.1) were identified exclusively in the rodent species *A. agrarius*.

### 3.3. Occurrence of VBZDs in Mites

A total of 6785 mites, grouped into 312 pools, were collected from 540 captured rodents distributed across 16 local regions (Table 3). The pathogens detected included SFTSV, present in 1.0% of pools (3/312, 95% CI: 0–2.0), *B. afzelii* in 0.3% (1/312 pools, 95% CI: 0–0.9), and *O. tsutsugamushi* also in 0.3% (1/312 pools, 95% CI: 0–0.9). *C. burnetii* was not detected in any of the samples. Regionally, the prevalence of SFTSV was noted as 2.3% in Cheorwon (1/43 pools, 95% CI: 0–6.8), 3.6% in Yeoju (1/28 pools, 95% CI: 0–10.4), and 10.0% in Gimcheon (1/10 pools, 95% CI: 0–28.6). In Yeoju, *B. afzelii* was detected with a prevalence of 3.5% (1/28 pools, 95% CI: 0–10.4), and *O. tsutsugamushi* showed a prevalence of 9.1% in Geoje (1/11 pools, 95% CI: 0–26.1). Seasonally, the prevalence of SFTSV significantly increased in the spring, reaching 2.7% (3/113 pools, 95% CI: 0–5.6, *p*=0.0211), with detection rates for *B. afzelii* and *O. tsutsugamushi* at 0.9% each during the same season (1/113 pools, 95% CI: 0–2.6). Despite similar numbers of rodents collected in the spring (265) and autumn (275), the chigger index of mites (number of collected mites/number of collected rodents) in autumn was 1.6 times higher than in spring, with values of 15.4 and 9.6, respectively. Notably, although one mite pool tested positive for *B. afzelii*, the corresponding rodent was negative for this pathogen.

### 3.4. Molecular and Phylogenetic Analyses

**Figure 2:**
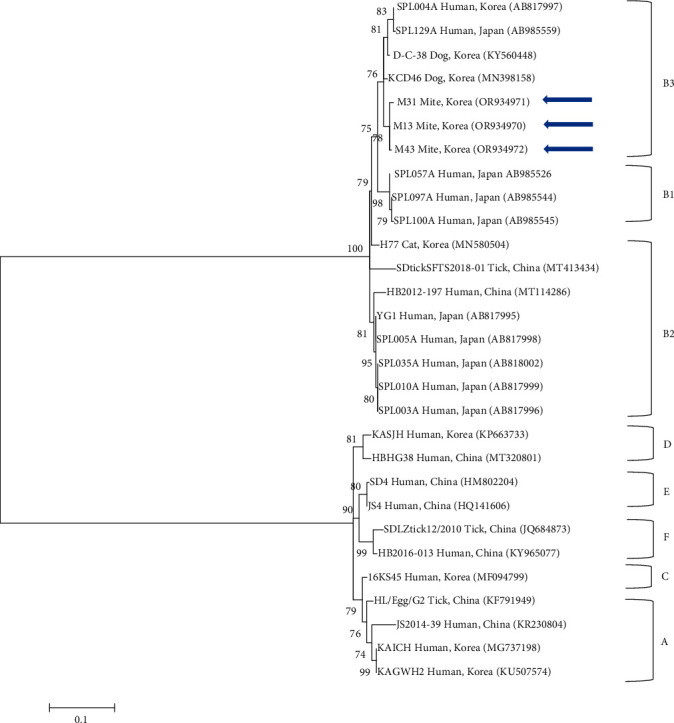
A phylogenetic tree constructed using the maximum likelihood method, based on the S segment sequences of SFTSV, is presented. Sequences analyzed in this study are highlighted with blue arrows. GenBank accession numbers for the referenced sequences are provided adjacent to their respective sequence names. Bootstrap support values from 1000 replicates are indicated at the branches, and the scale bar illustrates the phylogenetic distance. SFTSV, severe fever with thrombocytopenia syndrome virus.

Phylogenetic analyses revealed that the S segment sequences of SFTSV clustered with previously documented sequences, forming six distinct groups (A, B, C, D, E, and F) (Figure 2). Specifically, our three sequences were assigned to type B, subtype B3, exhibiting 96.2%–99.7% similarity, and sharing 97.2%–98.3% identity with the previously reported SFTS B-type isolates from GenBank. The sequences identified in this study have been deposited in GenBank under the accession numbers OR934970–OR934972 (SFTS S segment).

**Figure 3:**
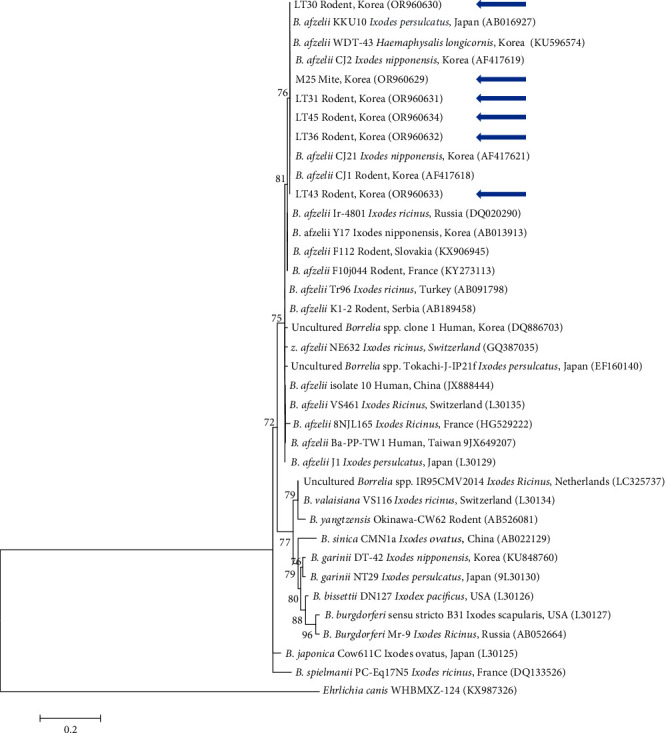
A phylogenetic tree constructed using the maximum likelihood method, based on the 5S−23S rRNA sequences of *Borrelia* spp., is shown. Sequences analyzed in this study are highlighted with blue arrows. GenBank accession numbers for the remaining sequences are presented adjacent to their respective sequence names. *Ehrlichia canis* served as the outgroup. Bootstrap support values from 1000 replicates are indicated at the branches, and the scale bar represents the phylogenetic distance. rRNA, ribosomal RNA.

For *B. afzelii*, the six 5S–23S rRNA sequences identified in both rodents and mites were classified as *B. afzelii* (Figure 3). These sequences demonstrated 99.6%–100% similarity, sharing 98.8%–100% identity with the previously documented *B. afzelii* isolates in GenBank. The sequences identified in this study have been submitted to GenBank under the accession numbers OR960629–OR960634 (*B. afzelii* 5S−23S rRNA).

**Figure 4:**
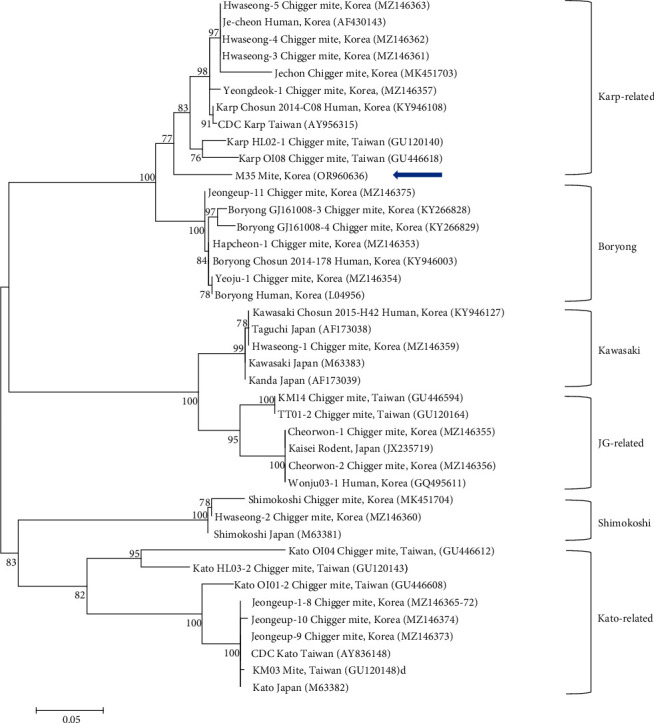
A phylogenetic tree constructed using the maximum likelihood method, based on the 56-kda sequences of *Orientia Tsutsugamushi*, is depicted. Sequences analyzed in this study are highlighted with blue arrows. GenBank accession numbers for the remaining sequences are provided adjacent to their respective sequence names. Bootstrap support values, based on 1000 replicates, are indicated at the branches, and the scale bar represents the phylogenetic distance.

Concerning *O. tsutsugamushi*, phylogenetic analysis showed that the 56-kda gene sequence from our study clustered with previously documented sequences into six groups: Karp-related, Boryong, Kawasaki, JG-related, Shimokoshi, and Kato-related (Figure 4). In mites, one sequence was classified within the Karp-related group, exhibiting a similarity of 96.4%–98.4% compared with other Karp-related sequences in GenBank. This sequence has been deposited in GenBank under the accession number OR960636 (*O. tsutsugamushi* 56 kDa).

## 4. Discussion

Climate change is exacerbating the density and distribution of vectors, thereby increasing the prevalence of VBZDs and altering ecological environments. This elevation in vector activity enhances the risk of domestic outbreaks, highlighting the urgent need for effective monitoring of vectors and VBZDs, as well as the development of comprehensive control strategies [2]. Furthermore, wild rodents, serving as reservoirs for various zoonotic pathogens, necessitate detailed investigations into their infections and the vectors associated with them. It is also essential to evaluate their roles in VBZD transmission. While international studies have identified rodents as carriers of pathogens responsible for SFTS [4], Lyme disease [5–7], and Q fever [8–10], research on other VBZDs in domestic rodents remains sparse, with the exception of significant findings on scrub typhus [11–13]. Despite ongoing detection of VBZDs in patients, rodents, and vectors globally, related studies are notably limited in South Korea. Therefore, it is crucial to analyze vectors, such as mites, and their associated diseases. Notably, cases of concurrent infections involving scrub typhus and SFTS have been reported in Myanmar [23], while simultaneous infections of scrub typhus with SFTS [24–27] and Q fever [28] have been documented in South Korea, indicating the potential for other mite-mediated VBZDs that warrant further investigation.

SFTS, caused by SFTSV, was first identified in China in 2011 amidst an outbreak of an unexplained illness [29]. In South Korea, the presence of SFTS has been documented in various hosts: in ticks (*H. longicornis*) at a rate of 2.1% (63/2973 pools) in 2020 [21], in wildlife at 32.3% (21/65) in 2013 [30], in dogs at 0.2% (1/426) in 2016, and in cats at 0.5% (1/215) in 2016 [31]. *H. longicornis* is recognized as the predominant species and the primary vector for SFTSV in South Korea [32–34]. Although SFTSV is predominantly observed from May to September, with prior data indicating a peak in positive detections in ticks during the summer months [34, 35], the mites analyzed in this investigation showed a notably higher rate of infection in the spring rather than in the summer. While SFTSV has been detected in mites globally [36], this study marks the first discovery of the virus in mites in South Korea. Despite global reports of coinfections of SFTSV with other pathogens in humans, no evidence of mite infections has been documented previously. The identification of SFTSV in mites in this study highlights the potential risk of mite-to-human transmission, underscoring the need for ongoing surveillance and effective pest management.

Lyme disease, caused by *Borrelia burgdorferi* sensu lato, is marked by an annual incidence exceeding 60,000 cases in Europe and 300,000 cases in the United States [37]. Since its initial identification in two patients in 2011, South Korea has reported ~20 cases annually [1]. *B. burgdorferi* and *B. garinii* were first isolated from rodents and ticks in 1992 [38]. Antibodies to *Borrelia* spp. have been identified in dogs [39, 40] and horses [41] as well as in ticks that feed on both domestic and wild animals [22, 42]. Our study found that among seven rodent species, *A. agrarius* was the most prevalent; it was also the only species in which VBZDs, specifically *B. afzelii*, were detected. Rodents were collected from five distinct habitats, with waterways and forests being the most common collection sites. However, the prevalence of *Borrelia* spp. was found to be higher in grasslands at 2.2% (2/92), compared to 1.4% (1/73) in rice paddies, 0.8% (1/128) in forests, and 0.8% (1/133) in waterways. This pattern suggests that despite lower collection frequencies, grasslands exhibit a higher *Borrelia* spp. prevalence. A similar trend was observed in Germany, where, although more rodents were collected from forests, the prevalence of *Borrelia* spp. was higher in grasslands at 13.5% (51/377) compared to 11.3% (81/717) in forests [43]. Consequently, the prevalence of *Borrelia* spp. demonstrates significant variability across different environments, with a noted predisposition for grasslands. In mites, *B. afzelii* was identified in only one pool. Previous studies have documented the presence of *Borrelia* spp. (not associated with relapsing fever or Lyme disease) in mites [44]. In this study, however, *B. afzelii*, the causative agent of Lyme disease, was found in mites for the first time ever. Notably, *B. afzelii* infections were detected in various rodents and mites, underscoring the necessity for further research to elucidate the relationship between these infections in both hosts. Moreover, it is crucial to conduct comprehensive nationwide studies on the prevalence of zoonotic infections and VBZDs, accompanied by comparative analyses across vectors, animals, and humans. Such research is vital for predicting disease outbreaks and for the establishment of an effective surveillance and rapid response system.

Q fever, induced by *C. burnetii*, typically presents as an acute febrile illness. In South Korea, infections by *Coxiella*-like bacteria have been reported in domestic ticks from horses [17], and ongoing infections have been observed in pigs [16], cattle [45], and goats [46]. Additionally, a 2011 study found *C. burnetii* infections in 18% (66/370) of rodents in China [47], and a study in Kenya reported a 5.5% (21/380) prevalence of *C. burnetii* in ticks [48]. However, despite these findings and the known instances of *C. burnetii* in rodents and ticks internationally, the current study did not identify any positive cases of *C. burnetii* infection. Given the potential for continued *C. burnetii* infections, ongoing monitoring and the development of management strategies are imperative.

Scrub typhus, a febrile illness transmitted by the larval stage of chigger mites infected with *O. tsutsugamushi* [49], has been documented annually in South Korea since its first reported case in 1951 [50]. Recent trends suggest that the distribution of *O. tsutsugamushi* may be influenced by climate change. To explore the effects of meteorological factors on its spread, our study analyzed the monthly incidence of scrub typhus from 2006 to 2012 in the temperate Laiwu district, China. The analysis showed that increases in monthly average temperature, relative humidity, and precipitation correlated with higher incidences of the disease. These factors were identified as key determinants in the transmission of *O. tsutsugamushi* [51]. In a specific case within our study, *O. tsutsugamushi* was detected only in a sample from the southern region of Geoje. Echoing the patterns observed in China, our findings suggest a possible correlation between the prevalence of scrub typhus and higher temperatures. This pattern suggests that *O. tsutsugamushi* may have a higher positivity rate in warmer southern regions. With global temperatures rising, this underscores the importance of continuous disease surveillance and preventive measures to combat the accelerated spread of *O. tsutsugamushi*.

Phylogenetic analysis categorizes SFTSV into six genotypes (A–F), according to a methodology previously applied in China [52]. In South Korea, the majority of SFTSV isolates (69.2%) was identified as genotype B, which is further divided into subgenotypes B1, B2, and B3. Subgenotype B2 was the most prevalent (36.1%), followed by B3 (21.1%) and B1 (12.0%) [53]. Although not widely reported, sequences similar to those of genotype B, typically found in ticks or humans, have also been identified in mites. Specifically, our sequences fall under subtype B3. The genus *Borrelia* includes several species such as *B. afzelii*, *B. valaisiana*, and *B. garinii*, with our sequence matching *B. afzelii*. Moreover, *Borrelia* spp. have been detected in various hosts, including humans and rodents, as well as in vector mites. Among the *O. tsutsugamushi* sequences, the 56-kDa gene indicated at least six distinct group variations, corresponding to strains from different regions. Our analysis classifies our sequence within the Karp-related group.

## 5. Conclusions

This study conducted a comprehensive nationwide survey to assess the regional distribution of rodents and mites by utilizing molecular analyses of four VBZDs. The findings demonstrated exclusive positivity for SFTSV and *O. tsutsugamushi* in mites, while *B. afzelii* was detected in both rodents and mites. Notably, this research documented the first domestic occurrence of SFTSV in mites. Furthermore, *B. afzelii* was identified in mites for the first time globally. These results highlight the urgent need for further analysis and investigation of VBZDs to predict future trends and to develop a monitoring system that aligns with these projections. Moreover, in South Korea, additional regional and ecological studies on rodents and mites are imperative to enhance the understanding of the risks associated with other VBZDs.

## Figures and Tables

**Table 1:** Primers employed for the detection of VBZDs.

Target disease		Primers	Target genes	Primer sequence (5′–3′)	Size (bp)	Reference
Scrub typhus	First	OT-F	56 kda	GCAATATTGCTAGTGCAATGTCTGC	1158	[14]
		OT-R		ATGCATGCATGRCGCTKCAATTTA		
	Second	OT-nF		ATAGGCCTATAAGTATWGCKGATCG	448–471	
		OT-nR		CATCTAGAYGCACTATTAGGCAAA		

SFTS	First	Np2F	S segment	CATCATTGTCTTTGCCCTGA	461	[15]
		Np2R		AGAAGACAGAGTTCACAGCA		
	Second	Nn2F		AAYAAGATCGTCAAGGCATCA	346	
		Nn2R		TAGTCTTGGTGAAGGCATCTT		

Q fever	First	Cox16F1	16S rRNA	CGTAGGAATCTACCTTRTAGWGG	1321–1416	[16, 17]
		Cox16R2		GCCTACCCGCTTCTGGTACAATT		
	Second	Cox16F2		TGAGAACTAGCTGTTGGRRAGT	719–813	
		Cox16R2		GCCTACCCGCTTCTGGTACAATT		

Lyme disease	First	Bb23S3	5S−23S rRNA	CGA CCT TCT TCG CCT TAA AGC	412	[18–20]
		Bb23Sa		TAA GCT GAC TAA TAC TAA TTA CCC		
	Second	Bb23S3nF		CTG CGA GTT CGC GGG AGA	226–266	
		Bb23SanR		TCC TAG GCA TTC ACC ATA		

Abbreviations: rRNA, ribosomal RNA; SFTS, severe fever with thrombocytopenia syndrome; VBZDs, vector-borne zoonotic diseases.

**Table 2:** Number of wild rodents collected and infected with *B. afzelii* in South Korea.

Species	Number of collected rodents (%)	Number of rodents infected with *B. afzelii* (%)
*A. agrarius*	439 (81.3)	5 (1.1)
*A. peninsulae*	5 (0.9)	0
*Craseomys regulus*	7 (1.3)	0
*Crocidura* spp.	64 (11.9)	0
*M. minutus*	21 (3.9)	0
*M. fortis*	3 (0.6)	0
*Mogera robusta*	1 (0.2)	0

Total	540	5 (0.9)

**Table 3:** Data collection based on regional and seasonal distribution and infection by VBZDs in wild rodent and mite samples in South Korea.

Group			Number of tested		Number of infected (%)					
			Rodents	Mites (pools)	SFTS		Lyme disease		Scrub typhus	
					Rodents	Mite pools	Rodents	Mite pools	Rodents	Mite pools
Regions	Northern	Cheorwon	67	819 (43)	0	1 (2.3)	2 (3.0)	0	0	0
		Gangneung	54	321 (21)	0	0	3 (5.6)	0	0	0
		Yeoju	43	636 (28)	0	1 (3.6)	0	1 (3.6)	0	0
		Hwasung	31	592 (27)	0	0	0	0	0	0
		Paju	46	955 (38)	0	0	0	0	0	0
	Central	Cheongju	24	339 (15)	0	0	0	0	0	0
		Boryeong	24	140 (7)	0	0	0	0	0	0
		Yesan	23	257 (12)	0	0	0	0	0	0
	Southern	Jeongeup	31	709 (28)	0	0	0	0	0	0
		Boseong	19	379 (15)	0	0	0	0	0	0
		Jinan	30	339 (18)	0	0	0	0	0	0
		Geoje	16	309 (11)	0	0	0	0	0	1 (9.1)
		Hapcheon	27	80 (7)	0	0	0	0	0	0
		Gimcheon	18	269 (10)	0	1 (10.0)	0	0	0	0
		Yeongdeok	26	448 (20)	0	0	0	0	0	0
	Jeju Island	Seogwipo	61	193 (12)	0	0	0	0	0	0

Seasons	Spring		265	2548 (113)	0	3 (2.7)	5 (1.9)	1 (0.9)	0	1 (0.9)
	Autumn		275	4237 (199)	0	0	0	0	0	0

Total			540	6785 (312)	0	3 (1.0)	5 (0.9)	1 (0.3)	0	1 (0.3)

Abbreviations: SFTS, severe fever with thrombocytopenia syndrome; VBZDs, vector-borne zoonotic diseases.

## Data Availability

Data supporting the conclusions of this article are included within this article. The newly generated sequences were submitted to the GenBank database under the accession numbers OR934970–OR934972, OR960629–OR960634, and OR960636. The datasets used and/or analyzed during the present study are available from the corresponding author upon reasonable request.
